# Vendors’ Perceptions and Experiences with WIC Online Shopping Implementation

**DOI:** 10.1016/j.cdnut.2024.102084

**Published:** 2024-01-27

**Authors:** Mayra Crespo-Bellido, Elizabeth Anderson Steeves, Jennie L Hill, Sarah Kersten, Allison Magness Nitto

**Affiliations:** 1 Gretchen Swanson Center for Nutrition, Omaha, NE, United States; 2 Division of Health System Innovation and Research, Department of Population Health Sciences, University of Utah, Salt Lake City, UT, United States

**Keywords:** WIC, nutrition assistance, online shopping, retailers, implementation science

## Abstract

**Background:**

Online shopping (OS) holds promise for improving the shopping experience for the Special Supplemental Nutrition Program for Women, Infants, and Children (WIC). However, little is known about vendors’ perspectives on implementing OS in the context of WIC.

**Objectives:**

The present study aimed to understand vendors’ experiences, needs, and barriers to WIC OS implementation.

**Methods:**

We recruited vendors at various stages of WIC OS planning and implementation (n = 16). Semistructured interviews were conducted, transcribed, and coded using subconstructs of the i-PARIHS framework domains (e.g., Characteristics of the Innovation, recipient, context, and facilitation) to assess determinants related to adoption and implementation of WIC OS among vendors.

**Results:**

Interviewees represented various organizations, including local (n = 5), regional (n = 4), and national (n = 5) entities, along with enablement platforms (n = 2). The interviews yielded themes related to experiences planning and implementing a WIC OS system (n = 7) and perceived needs and barriers (n = 3). Vendors drew on prior experiences with Supplemental Nutrition Assistance Program (SNAP) OS to inform WIC OS projects, stressing the importance of building relationships and collaborating, particularly in technical partnerships, during WIC OS implementation. They also highlighted the value of leveraging existing OS systems to implement WIC OS projects, discussed WIC OS perceived benefits, emphasized the role of educating staff and participants on its usage, and valued WIC OS implementation guidance provided by WIC agencies. Needs and barriers for vendors contemplating WIC OS implementation included the need for evidence of successful implementation of WIC OS projects, understanding current regulatory implications, and appraising existing priorities and financial considerations for adopting and implementing WIC OS.

**Conclusions:**

WIC OS innovations are integral to modernizing the federal food assistance program. The present study highlights the role of vendor engagement, collaboration, guidance from WIC agencies, and knowledge sharing in ensuring WIC OS success. These insights can inform how WIC State agencies engage vendors to implement WIC OS.

## Introduction

In 2022, the Special Supplemental Nutrition Program for Women, Infants, and Children (WIC) served ∼6.26 million low-income pregnant, postpartum, and breastfeeding women, infants, and children < age 5 [1]. This essential public health program provides participants with supplemental food, nutrition education, breastfeeding support, and referrals to immunizations and other health services [2]. WIC is administered at the state level by 89 WIC State agencies, including all 50 states, the District of Columbia, 33 Indian Tribal Organizations, and 5 territories [2] through federal grants provided by the United States Department of Agriculture’s Food and Nutrition Service (USDA-FNS). In addition to providing participants with essential nutrients needed for a healthy and balanced diet, research shows that participation in WIC leads to positive outcomes, including birth outcomes, feeding and diet practices, cognitive development, immunization rates, and reduced health care costs [3]. Yet, on average, 50% of eligible participants do not engage with the program [4], and among those who do enroll, many do not use all of the WIC food benefits available to them [5]. Solutions are needed to ensure that eligible individuals and families enroll in the program and, once enrolled, utilize the full value of their food benefits.

WIC participants use electronic benefit transfer (EBT) cards instore at WIC-authorized food vendors (e.g., grocery stores) where they can use their prescribed WIC food benefits [6]. Issues related to transportation to stores, difficulties locating specific WIC-approved foods, problems during the check-out process, and stigma over public benefit use present barriers that WIC participants experience while shopping for food instore [[7], [8], [9], [10]]. Prior research shows that online shopping has the potential to decrease instore shopping time and facilitate the identification and selection of WIC-approved items [11]. Offering WIC participants the option to shop for WIC-approved foods online helps create an equitable shopping experience for WIC and non-WIC participants. Early in the COVID-19 pandemic, an estimated 40% of United States households ordered their groceries online [12], and the recent expansion of the Supplemental Nutrition Assistance Program (SNAP) online shopping enabled SNAP households across all 50 States to utilize online shopping which potentially had an impact in reducing food insufficiency during this period [13,14]. Online grocery shopping has also increased in popularity among households with lower incomes. One study found in February 2023 that 27% of households with incomes of ≤$35,000 purchased ≥$50 worth of groceries in a national e-commerce platform that month [15]. Furthermore, research among participants of federal food assistance programs found online shopping increased access to healthy foods, such as fruits and vegetables [16], and one study found that most who shopped online during the pandemic planned to continue to shop online in the future [17]. Thus, WIC online shopping (WIC OS) has the potential to improve the WIC participant experience and reduce food access inequities when shopping with WIC food benefits [18]. Innovative projects led by WIC State agencies and vendor partners are underway to develop solutions for WIC OS and transactions through the WIC OS Grant, a multiyear 2-phase project funded through a cooperative agreement with USDA-FNS and the Gretchen Swanson Center for Nutrition (GSCN) [[19], [20], [21], [22], [23]]. Phase 1 involved a formative study to identify recommendations for implementing WIC OS, which led to the creation of a WIC OS resource guide called the Blueprint for WIC OS Projects, or “the Blueprint.” The ongoing Phase 2 involves 4 GSCN subgrant projects that received funding to implement WIC OS using the Blueprint as a guide [[19], [20], [21], [22]].

Some WIC Federal requirements may influence innovation and uptake of online shopping. For example, current regulations require food benefit transactions to occur in the presence of a cashier at a physical store (i.e., brick-and-mortar location) while not allowing for refunds and exchanges [24]. As part of the American Rescue Plan Act of 2021 (PL 117-2; ARPA), a United States federal response to the COVID-19 pandemic, USDA-FNS has temporary waiver authority to support WIC State agencies carrying out projects related to WIC outreach, innovation, and program modernization efforts, including WIC OS projects [25]. In addition, USDA-FNS released the WIC Online Ordering and Transactions and Food Delivery Revisions to Meet the Needs of a Modern, Data Driven Program proposed rule in February 2023 aimed at removing regulatory barriers to implementing WIC OS. USDA-FNS intends for this proposed rule to improve the WIC shopping experience while increasing equity and access to nutritious foods for WIC participants, thus positively impacting nutrition security [26].

WIC-authorized vendors are the cornerstone of the WIC participant experience, and engagement and support from vendors are essential for the successful use of WIC food benefits. Creating an online shopping experience suitable for WIC participants requires collaboration among WIC Federal, State, and local agencies, EBT payment processors, vendors, and other stakeholders. There are ∼40,000 WIC-authorized vendors in the United States as of 2023 [24]. Vendors must apply and receive approval from their WIC State agency to be WIC-authorized vendors, with authorization dependent on several factors. A key factor is that each State agency needs to authorize vendors separately compared with the authorization of a chain of vendors nationally [27]. Recent research demonstrates that identifying, engaging, and retaining vendors to implement WIC OS is challenging for WIC State agencies [19]. However, there is limited information available on vendors’ experiences and perceptions with WIC OS [11,[28], [29], [30], [31]]. Over the past 2 years, ARPA waivers and funding opportunities related to WIC OS have demonstrated their importance and potential; thus, it is imperative to understand the current perspectives of vendors, including their plans and experiences with adopting and implementing WIC OS. The purpose of this qualitative study was to explore the perceived ongoing facilitators and barriers to adoption and implementation of WIC OS for vendors given the flexibility in rules and opportunity for funding to support innovation in this area.

## Methods

### Overall study design, research questions, and conceptual framework

Ongoing process and evaluation efforts, including this substudy, occur throughout the WIC OS Grant to identify facilitators and barriers to the adoption and implementation of WIC OS. The overarching study design is a type 3 hybrid effectiveness implementation [32,33] approach using RE-AIM (*R*each, *E*ffectiveness, *A*doption, *I*mplementation, and *M*aintenance) and integrated Promoting Action on Research in Health Services (i-PARIHS) frameworks [[34], [35], [36], [37]]. i-PARIHS includes 4 primary constructs—Characteristics of the Innovation (e.g., WIC OS), contextual factors, recipient factors (i.e., implementers), and facilitation capacity [37]. It is hypothesized that organizations with high scores across these 4 constructs are more likely to be successful in adopting, implementing, and sustaining an innovation [37,38]. Vendors are key WIC OS implementation partners, and understanding the barriers and the potential facilitation needs of this group is essential; therefore, this qualitative substudy is grounded in i-PARIHS.

To inform ongoing and future WIC OS implementation efforts, the overarching research question of this substudy is: What are the factors that influence the vendors’ decision on whether to pursue WIC OS planning and implementation? More specifically, with the perspectives from these 3 subgroups of vendors, we aimed to answer the following subquestions: What are the perceived needs and barriers for planning and implementing a WIC OS system for vendors contemplating engaging in a WIC OS project? Among those planning or implementing a project, what actions facilitated vendors’ active engagement in a WIC OS project? What lessons have vendors learned from planning and implementing WIC OS projects that can inform vendors who are seeking to start their own projects?

### Recruitment

We recruited vendors at different stages of planning and implementing WIC OS projects. These included vendors that are contemplating planning or implementing WIC OS (subgroup 1), vendors that are implementing WIC OS outside of the GSCN subgrant project (subgroup 2), and vendors who are planning to implement WIC OS as part of the GSCN subgrant project (subgroup 3).

We used 2 mechanisms to identify potential interviewees for the first subgroup of vendors. First, we used the contact list of vendors gathered during Phase 1 formative study of the GSCN project [20], which included ∼30 unique vendors. Second, we worked with the WIC OS Grant Steering Committee, comprising WIC experts, to identify additional vendors who were interested in online shopping. The WIC OS Grant Steering Committee provided contact information for several vendors contemplating online shopping to improve the diversity of geographic regions and vendor size in our sample. For the second subgroup, we recruited vendors partnering with WIC State agencies involved in a project related to WIC OS outside of the GSCN subgrant. For the third subgroup, we recruited vendors participating in the 4 GSCN subgrant projects.

We contacted a total of 69 national, regional, and local unique vendors via email for an interview, and 16 completed the interview [overall response rate (RR) 23%]. Each vendor was sent 3 reminder emails at 1-week intervals requesting to schedule an interview. Vendors were classified as national if they offered services in most USDA-FNS Regions, regional if they offered services in multiple states within or across FNS Regions, and local if they only served one state.

### Interview guide development and measures

The semistructured interview guides were developed to align with i-PARIHS and tailored based on the vendor subgroup type (Table 1). Initial drafts were created by senior researchers with expertise in implementation science, WIC, and retailer engagement (JLH, AMN, and EAS). Drafts were reviewed and refined by the data collection team (MCB and SK). For vendors contemplating WIC OS (subgroup 1), we asked about perceived barriers and facilitators to implementing WIC OS and what it would take for them to offer online shopping to WIC participants. Vendors implementing WIC OS outside of the GSCN subgrant (subgroup 2) were asked to provide their experiences in planning, preparing, and implementing WIC OS and advice for recruiting and maintaining strong vendor relationships. Vendors planning to implement WIC OS with the GSCN subgrant project (subgroup 3) were asked about the successes, challenges, and advice for other vendors based on their experience thus far with the project.

**TABLE 1:** Sample questions from each interview guide

Subgroup 1—Vendors that are not planning or implementing WIC OS
• How feasible would implementing WIC OS be for [COMPANY NAME]? • What are the top 3 challenges or barriers to implementing WIC OS? • If [COMPANY NAME] was contemplating implementing WIC OS, what support would you need to do so? • If a funding opportunity becomes available to support WIC OS, would [COMPANY NAME] be interested?
Subgroup 2—Vendors implementing WIC OS outside of the GSCN subgrant
• Can you describe how WIC OS works for your store? • How do you ensure WIC participants have a positive online shopping experience? • What was the planning process to establish WIC OS? • What was the role of the WIC State agency in planning and implementing WIC OS services at [COMPANY NAME]?
Subgroup 3—Vendors planning to implement WIC OS with the GSCN subgrant project
• Tell me about [COMPANY NAME]’s experience with the WIC OS project so far? • What are the top 3 challenges or barriers to planning [and implementing] WIC OS? (Probe for: costs, staff, technology, other resources, leadership buy-in) • What advice would you give to other vendors planning to implement WIC OS? • What support would vendors need to provide WIC OS?

### Data collection

Two trained researchers in qualitative interviewing conducted the interviews online (MCB and SK). The interviews were conducted from February 2023 to April 2023, and each interview lasted ∼30 minutes. Interviews were voluntary, and vendors could choose not to answer a question or to end the interview for any reason without penalty. Upon completion, we offered vendors a $100 e-gift card to accept or donate to a charity of their preference. Interviews were audio recorded with permission from the vendor and transcribed verbatim by Rev (Austin, Texas, United States), a third-party transcription service. We achieved data saturation, defined as no new emerging themes in interviews [39], after completing 11 interviews and conducting 5 additional interviews to confirm saturation. Researchers checked all transcripts for quality assurance and removed any identifiable information from the transcripts. The University of Nebraska Medical Center Office of Regulatory Affairs determined the study to be exempt from human subjects review (Exemption #0013-22-EX).

### Data analysis

A senior researcher (JLH) adapted the i-PARIHS framework-based codebook developed by Ritchie et al. [35] and modified the codebook to operationalize subconstructs to provide a frame of reference relevant to a WIC OS project implementation (see Supplemental Table 1). For example, modifications to the codebook for this substudy reflect the WIC Program structure and identify the specific stakeholder or setting relating to the subconstruct (e.g., WIC-authorized vendors and their staff are recipients or internal context subconstructs can be within vendor organization, whereas outer context subconstructs are external factors such as WIC regulations). The adapted codebook was used to code a test transcript by 2 research team members trained in qualitative research (MCB and SK). Qualitative data were analyzed using NVivo 14 software [40] (Burlington, Massachusetts, United States). Intercoder reliability was calculated using NVivo’s coding comparison function, and a consensus process was used to determine that codes were clearly defined and could be consistently applied by the 2 coders. Research team members met to discuss coding discrepancies (e.g., nodes with a kappa coefficient < 0.7), reconciled differences through discussion, and refined definitions. The remaining transcripts were divided between 2 research staff members for coding (MCB and SK). Upon completion of coding all transcripts, researchers (MCB, SK, and AMN) identified major emerging themes (n = 10). Themes explaining the experiences of planning or implementing WIC OS projects were organized into 4 interrelated i-PARIHS domains.

**TABLE 2:** Vendors’ experiences with planning or implementing a WIC OS system

i-PARIHS Construct(s)	i-PARIHS Subconstruct(s)	Theme	Description	Example quote
Characteristics of the innovation	Underlying knowledge – local practice information Complexity	1. Vendors draw on prior experiences with SNAP online implementation to inform planning and implementing WIC OS.	Vendors in all stages of planning or implementing WIC OS, reported using their experiences with implementing SNAP online to predict facilitators and barriers with adapting current systems and processes to fit within the implementation of WIC OS. Additionally, vendors contemplating whether to implement WIC OS often compare the 2 programs and the business case for SNAP compared with WIC OS.	“The integration side of [SNAP OS] seemed like it took an extremely long time to get that in place. I want to say probably somewhere close to about 8 or 9 months before we actually, from start to finish, could offer SNAP online to our [DELIVERY SERVICE] customers, and we were one of the first ones to do that, so I'm sure they were working out the kinks in the system, but that makes it very challenging and ties up a lot of resources when everything is not worked out prior, and you're kind of the test for it. It takes up a lot of a company’s resources to do that.” – [Regional vendor contemplating WIC OS] “I think another [barrier] that we've kind of pushed for is the licensing because WIC scores our license individually. We would like to see WIC, like SNAP online, where it’s one online license, whether that’s nationwide or by each state, because it’s difficult to block off certain stores from pickup and delivery when we don't do WIC in store. […] The states we're partnering with within the pilot have worked with us on changing some of their requirements to get stores licensed so that most of our stores in their states can receive a WIC license to ease some confusion for customers.” – [National vendor planning WIC OS]
Recipients	Collaboration and teamwork	2. Vendors planning or implementing WIC OS mentioned that implementation requires relationship building and collaboration between all partners.	According to vendors planning/implementing WIC OS, finding working solutions requires open communication and collaboration between the private sector, government stakeholders, WIC State agencies, and vendors. This can help to build trust and foster a collaborative working relationship.	“We went through and worked out each step in the process for a customer, from creating an account to even after they received their order, if there are any issues. I think that has really helped us because there wasn't a ton of back and forth from all the different groups. We could all work collaboratively and all be at the table as we made these decisions. Then, on those calls, after we came to an agreement, we again worked through the flow to make sure it is exactly how everyone needed it to be to meet their development or what they could and couldn't do. I think that’s been very beneficial and has allowed us to, like I said, work collaboratively and really create that experience that meets everyone’s needs.” – [National vendor planning WIC OS]
Recipients	Personal attributes – values, beliefs, and goals	3. Most vendors interviewed discussed the perceived added value of WIC OS.	Vendors were motivated to consider, plan, or implement a WIC OS project because they felt that it would allow them to reach more WIC participants with structural barriers. For vendors planning or implementing a project, they also shared that implementing WIC OS continues to help them advance their technology and keep up with the changing technology environment.	“I’m just so happy to be involved in the project. I think it’s just so cool. It’s going to change the perceptions of WIC. […] And I would imagine participation’s going to increase, make it easier [for participants] to get food, and satisfaction will increase. I think it’s going to be a real inflection point for WIC and WIC participants.” – [Enablement platform, planning WIC OS] “I don't think there’s any lack of effort or desire on the part of WIC or the supermarkets to get this going. It’s just a question of if we can figure out the technology. I think everybody deserves equal access to food, so let’s figure out a way to do it.” – [Local vendor contemplating WIC OS]
Context characteristics	Networks and relationships	4. Vendors planning or implementing WIC OS leverage the expertise of technical partnerships when pursuing WIC OS.	In addition to partnerships with WIC State agencies, vendors emphasized the importance of leveraging technical expertise, such as IT departments and engaging with third-party processors to assist in adopting WIC OS	“I mean [a vendor planning WIC OS would] probably need a good IT partner to make it happen. It’s not easy. And I think that’s really the number one thing. Making sure you have some patience, you have a good crew, you put together a team that understands not just the WIC shopping experience, but also the backend at the store level.” –[Regional vendor, implementing WIC OS]
	Structures and systems	5. Vendors planning or implementing online shopping assess and leverage their existing online shopping systems and features to inform WIC OS.	Vendors with existing online shopping systems considered how current systems and existing features could integrate into WIC OS.	“[WIC customers] have the same experience that anybody else does…The system provides suggestions for complimentary items. I’m not sure about the development of additional information in this system for highlighting things as WIC-eligible, but typically, our customers who are shopping online are pretty clear about what is WIC-eligible, and they have a pretty seamless transaction…So, we already have these WIC customers who are ordering online. They’re just not paying online.”- [Local vendor implementing WIC OS]
Facilitation activities	Providing administrative and technical support	6. Vendors value guidance and support provided by Federal and State WIC agencies when planning and implementing WIC OS.	Vendors discussed the importance of receiving information surrounding program regulations, program requirements, and details on meeting program requirements from both State and Federal WIC agencies.	“They [WIC State agency] were really supportive and helpful, obviously, because it was during the pandemic. They wanted this option for their participants, and so they were open door and did whatever we asked and were really helpful.” – [Regional vendor implementing WIC OS] “And certainly they [WIC State agencies] had to serve these consumers, but they also cared about what we had to say because they knew how hard we were working as well to serve those consumers.” – [Local vendor planning WIC OS]
	Providing education/ information – marketing to participants Providing education/ information – educating staff	7. Vendors planning or implementing WIC OS discussed the importance of educating both store staff and WIC participants on utilizing WIC OS.	Providing sufficient education for participants utilizing WIC benefits online is a priority among vendors. Vendors noted the importance of having store staff adequately trained on the online shopping system to facilitate participants utilizing WIC OS.	“Well, a lot of it’s just making sure we train our team members to really focus on this transaction because we see it as a growth opportunity. So you really want to make sure that they have a good experience so they come back and use it. So it’s really making sure our team is just familiar with the rules so they feel comfortable helping customers with the process, because it’s not for everybody. We want to make sure that if a customer needs some help, that our folks can jump in there and give some help.” – [Regional vendor implementing WIC OS]

**TABLE 3:** Vendors’ perceived needs and barriers for planning, implementing, and maintaining a WIC OS system

i-PARIHS Construct(s)	i-PARIHS Subconstruct(s)	Theme	Description	Example quote
Characteristics of the innovation	Underlying knowledge – research and guidelines Underlying knowledge – patient needs, preferences, and experiences Complexity	1. Vendors contemplating planning or implementing need evidence of WIC OS implementation success to help inform their decision to engage in a WIC OS project.	Understanding the complete financial, staffing, and technological resources prior to investing in WIC OS is a priority among vendors. Similarly, proof of implementation success with WIC OS can facilitate vendors’ decisions to participate in WIC OS.	“I would say we would not be the leader in this at all. We would be a follower. As somebody comes out, potentially nationwide or multiple states, and can accept WIC online and is making it work, I would assume that there would have to be some changes made to help facilitate that or smooth that out, and that’s when we would potentially start looking at it. It wouldn't be until after somebody else rolled it out, if that makes sense. So, we are not going to dive into the development of this until standards have been set. – [National vendor contemplating WIC OS]
Context characteristics	Structures and systems Political factors and dynamics	2. Vendors contemplating planning or implementing WIC OS consider how current regulations will factor into developing a WIC OS system.	Vendors contemplating planning or implementing WIC OS assess how current online shopping systems will meet Federal WIC regulations. Additionally, vendors discussed the need for policies that support WIC OS and regulatory changes to advance the WIC Program.	“So that’s another challenge [with WIC] when we’re dealing with SNAP; it’s a really clear one set of national rules. But being able to build out 89 different variations on that and make sure that they’re getting updated daily with the changes to the approved products list. Those are all areas that just create additional complexity and drive incremental cost for us as a national online vendor to work through each one of those things.” – [National vendor contemplating WIC OS]
Context characteristics	Organizational priorities	3. Vendors contemplating planning or implementing WIC OS weigh existing priorities and the financial impact of WIC OS when deciding to pursue it.	Vendors noted competing organizational priorities as a challenge for implementing WIC OS. Similarly, the investment in WIC OS compared to the return on investment for the number of WIC participants served is a consideration for some vendors.	“Also, the challenge that we faced was that WIC represents a small percentage of a vendor’s sales, about 2% or 3%. So, I think that there was some hesitancy on their end because they didn't really want to make an investment for WIC because, in their mind, it didn't represent enough of their sales.” - [Enablement platform planning WIC OS] “And when you look at the number of WIC sales we did, it was significantly more than 10 times less than what we did for SNAP sales.” - [National vendor contemplating WIC OS]

## Results

The results section of this manuscript presents an overview of the study’s findings regarding vendor characteristics (Table 2), vendors’ experiences with WIC OS planning and implementation (Table 3), as well as the perceived needs and barriers associated with establishing and maintaining a WIC OS system shared by vendors’ contemplating implementation (Table 4). Example quotes are included in the theme tables TABLE 3, TABLE 4 below to support the themes.

**TABLE 4:** Responding vendor characteristics (n = 16)

Variable	Category	Frequency
Vendor classification	Local	5
	Regional	4
	National	5
	Sales enablement platforms^1^	2
USDA-FNS Regional Office Coverage^2^	Nationwide	6
	Multiregional	3
	Mountain plains	2
	Northeast	2
	Southeast	1
	Southwest	1
	Western	1
	Mid-Atlantic	0
	Midwest	0
Offering online shopping to customers	Yes	14
	No	2
Offering SNAP OS	Yes	11
	No	5
Vendor WIC OS stage	Contemplating WIC OS	10
	Planning WIC OS	4
	Implementing WIC OS	2

1 These 2 sales enablement platforms are not currently WIC-authorized vendors; however, they provided useful insights on potential facilitators and barriers of implementing WIC OS if authorized in the future.

2 Not mutually exclusive. Vendors could be located in 1 or more USDA-FNS Regions.

### Vendor characteristics

Of the 16 vendor representatives interviewed, 5 represented local vendors, 4 represented regional vendors, 5 represented national vendors, and 2 represented enablement platforms, i.e., entities that provide food from a virtual marketplace or an intermediary entity that uses their online shopping platform to facilitate the provision of food from a retailer, such as e-commerce platforms and other technology companies working to facilitate WIC online shopping (Table 4). Most vendors offered some version of online shopping, and two-thirds offered SNAP online shopping (SNAP OS) in particular. However, only 6 of the 16 interviewees were implementing WIC OS 4 were a part of the subgroup planning to implement WIC OS through GSCN subgrants, and 2 were part of the subgroup implementing WIC OS outside of the GSCN subgrants. The other 10 were only contemplating whether to implement a WIC OS project. The geographic coverage of the vendors spanned the United States based on the national and multiregional vendors interviewed. Five of the national vendors interviewed represent some of the largest vendors nationwide [41]. Representatives interviewed were all in decision-making roles within their organizations (e.g., owners, store managers, senior product managers, and marketing managers) and had extensive knowledge about the WIC Program regulations and implementation.

It should be noted that response rates varied by vendor characteristics and subgroup (e.g., vendors only contemplating WIC OS had an RR of 19%, vendors implementing WIC OS outside of the subgrant program had an RR of 20%, and vendors partnering in one of the subgrant projects had a RR of 67%). Respondents were more often national organizations (RR: 67%), offering some form of online shopping (RR: 31%) and implementing a WIC OS project (RR: 40%). Nonrespondents were more often from the USDA-FNS mid-Atlantic and midwestern Regional Offices (RR: 0% for both) or Southwest and multiregional (RR: 14% for both), from regional (RR: 16%) and local (RR: 23%) organizations and did not offer online shopping (RR: 17%).

### Vendors’ experiences with planning or implementing WIC OS

#### Characteristics of the innovation

The Characteristics of the Innovation domain of the i-PARIHS framework identify traits of the innovation (i.e., WIC OS systems) that enable or hinder vendors’ implementation. The innovation is defined as WIC OS or the system(s) necessary to support WIC OS; therefore, Characteristics of the Innovation domain subconstructs, such as complexity, underlying knowledge, or compatibility, all refer to vendor perceptions of the systems or adaptation of their current online systems that will be necessary to implement WIC OS. The most salient subconstructs in the vendor interviews included “underlying knowledge” (e.g., choosing or contemplating implementation of WIC OS based on existing evidence) and “complexity” (e.g., considerations about the complexity of WIC).

##### Theme 1: Vendors draw on previous SNAP online implementation experiences to inform WIC OS

One important theme was that vendors in different stages of contemplation, planning, and implementation of WIC OS reported using their experiences implementing the SNAP online pilots to predict their capacity for implementing WIC OS. Vendors drew on local practice information (e.g., experiences with SNAP online) to predict the level of complexity, facilitators, and barriers to implementing WIC OS. During SNAP OS implementation, existing partnerships and training and education plans facilitated the implementation process, which also could be applicable to supporting WIC OS implementation. However, the technical complexity of integrating in store technology with payment processor systems was identified as a significant barrier that could impede the adoption of WIC OS. WIC provides a prescriptive food benefit rather than a cash benefit like SNAP provides (e.g., WIC participants may receive a dozen eggs, a gallon of milk, and 18 ounces of cereal). This feature makes transacting WIC food benefits more complex instore, and that complexity is relevant for online shopping systems. Additionally, vendors contemplating WIC OS often compared the 2 programs and discussed the business case for implementing SNAP compared with WIC OS. Many vendors reported that the business case and larger customer base for SNAP OS were more compelling than the case for WIC OS. A national vendor contemplating WIC OS illustrated how SNAP OS implementation and the role of the business case factor in informing WIC OS in the quote, *“*Online SNAP is a lot easier to do than online WIC [and] that took us 2 years, so it’s all about the business case, to be quite honest.”

#### Recipients

The recipients domain encompasses the perspectives and characteristics of the individuals/entities involved in the WIC OS implementation process (i.e., vendors). The most salient subconstructs were “collaboration and teamwork” (e.g., the role of collaboration and teamwork in implementation) and “personal attributes” (e.g., vendors’ perspective on the added value WIC OS offers for WIC participants).

##### Theme 2: Vendors planning or implementing WIC OS mentioned implementation requires relationship building and collaboration

Vendors planning or implementing WIC OS discussed building a successful partnership between WIC State agencies and vendors as crucial and that effective communication is key to building trust and fostering a collaborative working relationship. Successful implementation of WIC OS requires a shared understanding of the objectives and goals of the project, as well as a willingness to work together to address any challenges that arise. These vendors suggested that regular meetings and ongoing dialog can facilitate effective communication between WIC State agencies and vendors. They recommended that WIC State agencies provide clear guidance and expectations for vendors and that vendors provide feedback and suggestions to the WIC State agencies. In summary, vendors’ collaborative teamwork and communication with WIC State agencies facilitate WIC OS implementation. For example, a local vendor engaging in 2-way communication with a WIC State agency while planning a WIC OS project noted, “I was very appreciative of the fact that they were seeking [feedback]. They really wanted to solve the issue as well.”

##### Theme 3: Most vendors understand the perceived added value of WIC OS

Overall, most vendors expressed positive views about the potential for WIC OS to address some of the structural barriers that prevent WIC participants from accessing healthy food. Vendors in different stages of contemplation, planning, or implementation felt that WIC OS would allow them to reach WIC participants in food deserts, rural areas, and those with transportation barriers. Additionally, vendors noted that WIC OS would benefit participants with time constraints due to childcare responsibilities. Furthermore, vendors planning or implementing WIC OS also identified benefits for themselves, noting that implementing WIC OS would allow them to advance their technology and keep up with the changing technology environment. By implementing WIC OS, vendors felt they would be better able to serve their customers, including those who prefer online shopping or those who may be unable to come to the store due to physical limitations. A local vendor interested in implementing WIC OS noted, “Being a rural area, we do have some of our customers that are 20 miles away, so if they can order, make sure we have it instead of driving all the way here and then finding out we're out of that product till tomorrow because the truck hasn't come yet, or something like that, I think that would be beneficial.”

#### Context characteristics

The Context Characteristics domain in the i-PARIHS framework includes internal and external factors related to implementation of WIC OS. Internal context can be within store or within chain/brand factors, whereas external context are factors external to an individual vendor that influence implementation, such as State or federal rules or regulations and broader industry-wide trends. The themes within this domain discussed the subconstructs of “networks and relationships” (e.g., existing networks and relationships with technical partners) and “structures and systems” (e.g., assessment of vendors’ online shopping systems).

##### Theme 4: Vendors planning or implementing WIC OS leverage the expertise of technical partnerships when pursuing WIC OS

In addition to collaboration with WIC State agencies, vendors noted that involving information technology (IT) subject matter experts and third-party processors (i.e., payment processor who provides transaction processing and operational services for vendors) in the planning and implementation stages helps ensure that the WIC OS system is technically feasible and compatible with existing systems. Vendors emphasized the importance of IT expertise in developing the WIC OS system to ensure that the system is user-friendly and easy to use for both vendors and WIC participants. In addition, vendors recommended engaging with third-party processors to facilitate the planning and implementation of WIC EBT transaction processes, ensuring that the vendor receives payment promptly and accurately. Vendors highlighted that involving third-party processors also helps vendors navigate the complex payment requirements associated with WIC transactions. The need to collaborate with all stakeholders’ IT departments in planning WIC OS is illustrated in this quote by a regional retailer: “Make sure you do have dedicated tech resources because it does take a lot of tech development, not just on our side, but also with each state, each processor.”

##### Theme 5: Vendors planning or implementing WIC OS assess and leverage their existing online shopping systems and features to inform WIC OS

Vendor perspectives on the implementation of WIC OS varied based on their existing online shopping systems. Vendors with existing online shopping systems considered how their current systems could integrate into WIC OS during planning and implementation stages. These vendors discussed utilizing existing features, such as partnering with third-party delivery services or using their own web-based applications when designing and implementing WIC OS systems. A local vendor implementing WIC OS found success with utilizing a third-party delivery service and indicated that “We also use [DELIVERY SERVICE]. Everything’s really going smoothly. We’ve done it long enough that the bugs are out of it for the most part.” In contrast, vendors without existing online shopping systems expressed concerns about the cost and feasibility of implementing WIC OS. These vendors were uncertain about the technological requirements for online shopping and the potential financial burden of implementing a new system. Some alternatives mentioned for vendors without online systems included partnering with third-party vendors to develop vendors’ systems or contracting with existing enablement platforms that could facilitate WIC OS.

#### Facilitation activities

The themes most closely related to the Facilitation domain of i-PARIHS discussed the WIC OS stakeholder activities that enabled implementation. Facilitation is currently underway for a few WIC OS projects; however, this information may be useful to inform other future facilitation activities that would support vendor implementation of WIC OS. Vendors discussed the subconstructs “providing administrative and technical support” and “providing education/information” as facilitating WIC OS implementation processes.

##### Theme 6: Vendors value guidance and support provided by Federal and State WIC agencies when planning and implementing WIC OS

Federal and State WIC technical support for vendors was discussed as an important facilitation activity. Vendors emphasized that when WIC State agencies provide essential information on program regulations and requirements, it streamlines WIC OS planning and implementation. Moreover, vendors noted the continued need for federal guidance to ensure that national vendors can meet WIC requirements. Vendors contemplating implementing WIC OS indicated that a lack of clear guidelines could create confusion and slow the implementation process, negatively affecting their willingness to implement WIC OS in the future. For national vendors contemplating WIC OS implementation, federal support could help vendors navigate the differences in regulations across states, as illustrated by the quote: “It would be much easier if we did have some federal guidance on WIC and not so much as from a State-to-State basis […] if you are a larger retailer, it may get very complicated having to make sure you’re following [different States’ WIC regulations].”

##### Theme 7: Vendors planning and implementing consider the importance of educating both store staff and WIC participants on using WIC OS

Vendors indicated the importance of promoting WIC OS services among WIC participants and educating staff on how to help WIC participants navigate online shopping as Facilitation strategies to implement WIC OS. Vendors identified that many WIC participants might not be familiar with the online shopping system and, therefore, may require advertising and additional support to navigate the system. Vendors suggested that providing clear and concise instructions for WIC participants and ensuring that the online shopping system is user-friendly and accessible can help facilitate the use of WIC OS. In addition to WIC participant education, vendors also emphasized the importance of adequately training store staff on the online shopping system. Vendors noted that store staff play a critical role in supporting WIC participants who are utilizing WIC OS; thus, it is essential that staff members are knowledgeable about the WIC OS process. Vendors suggested that vendors providing instore training sessions for store staff and having a designated staff member available to assist with WIC OS orders can help ensure that WIC participants receive the support they need. A regional vendor implementing WIC OS discussed the importance of having staff be comfortable with assisting WIC participants by stating, “So it’s really making sure our team is just familiar with the rules, so they feel comfortable helping customers with the process because it’s not for everybody. We want to make sure that if a customer needs some help, our folks can jump in there and give some help.”

### Vendors’ perceived needs and barriers for planning, implementing, and maintaining a WIC OS system

The emerging themes for contemplating vendors’ perceived needs for planning, implementing, and maintaining a WIC OS system were organized into 2 of the salient i-PARIHS domains: Characteristics of the Innovation and Context Characteristics.

#### Characteristics of the innovation

On the Characteristics of the Innovation domain, vendors discussed the need for clear guidance and an understanding of WIC participants’ needs, preferences, and experiences that were best captured by the “underlying knowledge” subconstruct.

##### Theme 1: Vendors contemplating planning or implementing need evidence of WIC OS implementation success to help inform their decision to engage in a WIC OS project

Throughout the interviews, vendors contemplating whether to implement WIC OS noted the need for guidance and evidence from other vendors who have successfully planned for and implemented a WIC OS system. Ensuring a comprehensive understanding of the financial, staffing, and technological resources required for WIC OS is crucial for vendors to decide whether to implement WIC OS. Similarly, for implementation of WIC OS, vendors sought clarity regarding specific technical processes for a WIC OS system, such as how to handle substitutions. Ultimately, vendors were interested in understanding how WIC participants will be impacted by WIC OS. Vendors identified needing evidence-based guidelines for ensuring that a positive shopping experience is equally feasible for WIC participants who utilize WIC OS compared to non-WIC participants. A quote by a national vendor contemplating WIC OS illustrates this theme, “We're still trying to just get enough meat on the bones, to feel like we have higher confidence around what an implementation really should look like and what the best practices would be around the customer experience for that. And as soon as we could nail those things down, we'd be in a much stronger position to move forward.”

#### Context characteristics

Within the Context Characteristics domain, vendors discussed the subconstructs of “structures and systems,” “political factors and dynamics” (e.g., State and federal WIC regulations), and “organizational priorities.” Overall, vendors indicated that they need to factor in regulations and organizational priorities when deciding whether to implement WIC OS.

##### Theme 2: Vendors contemplating planning or implementing WIC OS consider how current regulations will factor into planning or implementing a WIC OS system

Vendors contemplating WIC OS consistently mentioned the uncertainty of how current WIC regulations would impact planning and implementing a WIC OS project. Specifically, these vendors sought to understand how their structures and systems could facilitate online shopping while meeting existing WIC regulations and guidelines. Vendors specifically were interested in understanding how to keep an accurate inventory to meet regulatory standards of WIC-approved items across multiple states, how vendors in multiple states can navigate adapting their payment systems while still meeting WIC Program standards, and continuing to assess and expand allowable WIC items to reduce WIC participant confusion among what can and cannot be purchased with WIC benefits. Vendors highlighted that continuing to modify WIC Program regulations to make WIC OS more viable can help create conditions that are necessary for planning and implementing a WIC OS system. A national vendor contemplating WIC OS noted, “One of the big ones...is regulatory change. The current regulatory landscape is not really attributable to an online experience.” For example, current regulations require WIC transactions to occur in the presence of a cashier, which was more relevant to the previous paper voucher system that was used by WIC to provide benefits prior to the role of WIC EBT (Currently, requests can be made to USDA-FNS via the ARPA waiver process for flexibility with this requirement. https://www.fns.usda.gov/wic/transaction-without-presence-cashier). This is illustrated by the following quote: “We're missing some of our customers. […] We'd like to be able to deliver to them and reach a population that may be either homebound or that doesn’t have reliable transportation. And we're not able to do that at this time because the WIC customer must be present and come inside to pay.” However, vendors contemplating WIC OS are optimistic that with the right support (e.g., clear guidance on regulatory change), they can navigate the regulatory landscape and successfully offer WIC OS services that meet the needs of WIC participants while ensuring compliance with federal regulations.

##### Theme 3: Vendors contemplating planning or implementing WIC OS weigh existing priorities and the financial impact of WIC OS when deciding to pursue WIC OS

Vendors expressed the importance of assessing existing priorities as a deciding factor for engaging in WIC OS. Vendors explained that although planning for implementing WIC OS as an investment is on the horizon, due to limited information surrounding WIC OS, it was not a high priority among vendors currently. For example, one vendor indicated that “it [WIC OS] is recognized as one of the items that we need to do on our roadmap. It’s just not prioritized super high right now.” Similarly, whereas vendors emphasized the importance of serving their current WIC participants, they noted the financial return on WIC benefits was not a priority compared with other revenue streams, such as SNAP benefits.

## Discussion

We identified several key findings regarding vendors’ decisions to pursue WIC OS. Notably, vendors’ experiences with SNAP continue to influence their perceptions and decisions around WIC OS implementation. As more vendors continue to offer SNAP OS, the lessons learned, and technology developed could be leveraged when implementing WIC OS. Nevertheless, WIC-specific tailoring will be needed due to the programmatic differences between SNAP and WIC. Vendors contemplating WIC OS implementation also mentioned the difference in revenue between SNAP and WIC. Due to the eligibility requirements and food benefits allowed, SNAP will continue to generate more revenue than WIC for most vendors. Yet, it should be noted that 50% of WIC families also participate in SNAP [42], and vendors offering both SNAP and WIC online could help attract both SNAP and WIC participants.

### Factors impacting WIC OS adoption and implementation

The adoption of WIC OS might require additional time and resources among those vendors that do not currently offer SNAP OS, use third-party platforms to facilitate online shopping (e.g., Instacart), or do not currently offer any online shopping. In these cases, these vendors can leverage the support and experiences of their WIC State agency and EBT payment processor to begin discussions regarding WIC OS. External funding opportunities also could be beneficial for these vendors to support the planning and development of a WIC OS system. In addition, as mentioned by most vendors interviewed in this study, WIC OS is important to the overall customer service provided by the vendor and helps reduce barriers to food access for WIC participants. Vendors in the planning and implementation stages also mentioned that providing WIC OS is necessary to align with the current advancements in technology in the retail sector. WIC State agencies and other partners can communicate these insights when engaging vendors to adopt WIC OS.

Because WIC OS is a relatively new process, it is not surprising that some vendors are waiting for lessons learned and guidance from other vendors that implemented WIC OS. GSCN’s WIC OS Grant [23] will provide updated and additional guidance based on findings from the subgrant projects and other project activities that could be useful for vendors interested in WIC OS. The USDA-FNS proposed rule [26] for WIC OS also could help alleviate some vendor concerns regarding the future of WIC OS; thus, as the federal rule process proceeds and is made final, more vendors might feel more comfortable investing staff and resources toward WIC OS.

As vendors continue to adopt WIC OS, more research and published guidelines for its implementation will become available. Meanwhile, WIC-authorized vendors in this study reported using SNAP OS implementation experiences to guide their thoughts on adopting, planning, and implementing WIC OS. A recent study showed vendors implementing SNAP OS recognized the benefits for customers (especially for mothers with young children) and high acceptability from tech-savvy groups (e.g., younger adults, individuals with families, and people with disabilities) [31]. The WIC-authorized vendors contemplating WIC OS interviewed for the current study also reported similar perceived benefits of WIC OS but also expressed concerns about the uptake of online shopping among WIC participants and wished to see more evidence before adopting WIC OS. For example, one regional WIC vendor shared that they wanted to know how many participants used WIC OS in pilot projects before implementing it in their store. Notably, whereas vendors implementing SNAP OS reported challenges with order fulfillment, staffing, and customer ordering barriers [31], vendors interviewed in this study discussed regulatory concerns and lack of clarity regarding the financial impact of implementing WIC OS as the main barriers to adoption. Funding opportunities, such as grants, could be utilized to help assist WIC State agencies and vendors in establishing or making WIC-specific updates to their online shopping systems.

When discussing facilitators, vendors planning and implementing WIC OS expressed the value and importance of partnerships to the success of the projects. Key partners noted were WIC State agencies, the federal government, and IT experts. This aligns with previous research on the entities to be involved with the WIC OS process [20]. It is helpful to involve these partners as early as possible in the WIC OS planning process and to engage in frequent communication and information sharing on the requirements and needs of each partner related to WIC OS.

### Future of WIC OS research and implementation

Future research could investigate the usability and accessibility of WIC OS systems from the WIC participant perspective, as there are few studies to date that have done this in WIC [11,43]. These small-scale studies assessed WIC participants’ perceptions of WIC OS but did not test and assess systems with online transactions with WIC participants. They were conducted before the COVID-19 pandemic. However, findings from these studies regarding participant uptake align with the findings from this study about the importance of WIC participant awareness of WIC OS. As noted by vendors in this study, ensuring that WIC participants are aware of and able to navigate WIC OS systems is critical to the success of WIC OS and ultimately achieving the goal of increasing food access for WIC participants. Future research also could assess best practices for training retail staff in fulfilling orders and supporting WIC participants with WIC OS. Retail staff knowledge of the WIC policies and processes and how policies and processes may differ online compared with instore are important factors for vendors to incorporate when planning and implementing WIC OS; this has been cited as a concern for vendors in similar studies [31]. Another key area of future research could focus on assessing lessons learned when vendors expand WIC OS to additional locations within and to other states and regions. The research also could identify any cost and time efficiencies as part of these expansion activities by tracking start-up and ongoing costs of WIC OS, as this information may be essential for vendors with multiple locations as they decide to adopt WIC OS.

The future of WIC OS also could be influenced by the USDA-FNS final rule after public comments are considered on the proposed rule [26]. One notable area is the potential authorization of internet vendors as WIC-authorized vendors. Although our study did not interview a large sample of internet vendors due to the primary focus on WIC-authorized vendors, the limited information gathered indicates that internet vendors are interested in WIC OS. The potential reach of these vendors nationwide is promising to increase WIC participants’ access to WIC food benefits, including WIC participants residing in food deserts [44]. For instance, one large vendor with online and store locations reported nearly all SNAP households have access to using SNAP benefits online, which is consistent with USDA estimates that 99% of SNAP participants have access to shopping online [13,45]. In anticipation of the USDA-FNS WIC OS final rule and its potential impact, participants discussed the proposed rule related to online shopping in this study; however, other federal rules processes, such as the WIC Food Package revision, are also underway [46] and it is not known how these changes may impact vendors’ willingness to obtain and maintain WIC-authorized status. However, both initiatives highlight the importance of Federal and State support to vendors, as vendors play a critical role in the overall success of WIC and its role in improving healthy food access for millions of American families.

### Study strengths and limitations

A key strength of the study was the utilization of a robust implementation science framework to support the interview guide development, codebook adaptation, and analysis [35]. The i-PARIHS constructs and subconstructs enabled us to identify and classify key themes related to WIC OS project implementation. WIC OS is a complex process that depends on commitment and collaboration across multiple entities and requires a systems-based approach to understand the friction points that hinder adoption and implementation. As the WIC OS Grant progresses, ongoing sub-studies and evaluation activities will identify challenges and potential solutions and inform the development of best practices to support the widespread adoption and implementation of WIC OS.

Another significant strength of this study is that it provides the perspective of vendors. There is a dearth of literature that examines this critical perspective when examining federal nutrition programs and food access initiatives. Further, most studies that report vendor perspectives are limited to small, independent retailers that do not represent the larger regional and national corporations that provide a significant proportion of food to communities [31,[47], [48], [49], [50]]. This study includes a diverse group of vendors based on size and geographic location, including some of the largest vendors in the United States that reach millions of families nationwide. Local, regional, and national vendors all play a critical role in providing food access to WIC participants; thus, their buy-in and willingness to adopt and implement WIC OS is essential.

This study also has limitations. Although we achieved thematic saturation with 16 completed interviews, the findings may not be generalized to represent all vendors. A challenge to recruitment stemmed from having only generic contact information for the recruitment email, which may have decreased the likelihood of response from some of those vendors. We engaged in several strategies to improve recruitment (e.g., requested decision makers’ contact information from national professional organizations for underrepresented geographic regions). Response rates varied drastically by vendor characteristics; regional vendors had the lowest response rates compared with local and national vendors, and vendors not offering any online shopping often declined interviews, citing that their insights were not relevant to the project. Notably, we were unable to interview vendors specific to the Midwest and mid-Atlantic Regions; however, higher response rates from national vendors may have helped improve geographic coverage. We also experienced initial challenges with identifying who was the most appropriate person to interview within each retail company; however, we included probes as part of the recruitment process to help identify those involved in the decision-making process for WIC OS. Lastly, WIC OS is a relatively new endeavor, so there are likely experiences that vendors have not faced yet related to WIC OS. Therefore, additional follow-up interviews could be warranted.

In qualitative research, it is important to recognize and acknowledge how the experiences, beliefs, relationships, and positionality of the researchers may influence the research process [51]. This research team has extensive experience in WIC research and is currently leading a USDA-funded effort to promote online shopping with WIC. Related to this, the research team had previous relationships with some of the interviewees prior to conducting this substudy, which may have influenced the interviewees’ willingness to participate in the interviews and the types of information they would share.

In conclusion, this is the first study, to our knowledge, that assesses the vendors’ perspectives and experiences with WIC OS implementation. The findings provide useful insights into barriers and challenges for implementation and potential strategies to engage and support vendors with WIC OS. The study emphasized that the success of WIC OS is based on commitment and collaboration from WIC vendors with thorough and timely guidance from Federal and State agencies and continuous sharing of lessons learned from vendors at each stage of the WIC OS process. These findings are essential to developing useful guidance to help WIC State agencies engage vendors as important partners in the implementation and long-term success of WIC OS.

### Author contributions

The authors’ responsibilities were as follows – AMN and JLH: designed the research and provided study oversight. MC-B and SK: conducted research and analyzed the data. MC-B, SK, and AMN: drafted the manuscript. AMN, JLH, and EAS: reviewed the manuscript and provided subject matter expertise, and all authors: read and approved the final manuscript.

### Conflict of interest

The authors report no conflicts of interest.

### Funding

Supported by the Food and Nutrition Service, the US Department of Agriculture.

### Data availability

Data described in the manuscript, code book, and analytic code will not be made available to protect the confidentiality of the interviewees.

## Appendix A Supplementary data

Multimedia component1 Multimedia component1
