# Supplementary material for: Vendors’ Perceptions and Experiences with WIC Online Shopping Implementation
Source: Curr Dev Nutr. 2024 Jan 27;8(2):102084. doi: 10.1016/j.cdnut.2024.102084 (PMC10875264; doi:10.1016/j.cdnut.2024.102084)
Supplement: Multimedia component1 [file mmc1.docx]

| **Supplemental Table 1. Examples of i-PARIHS constructs and subconstructs in modified codebook and tailored for WIC OS.** | |
| --- | --- |
| i-PARIHS Constructs | i-PARIHS Subconstructs |
| Characteristics of the Innovation | Evidence: Underlying sources of knowledge about the effectiveness or other evidence for WIC OS.  Evidence: Local practice or industry knowledge based on vendor experiences.  Clarity: Degree to which the process of implementing WIC OS is understood, including specifics of what components must be implemented and what can be adapted or changed.  Complexity: Ways in which the process of implementing WIC OS is simple or complicated. |
| Recipients | Personal Attributes – Values, Beliefs, and Goals: Personal traits or characteristics of the vendor’s staff/decision makers.  Collaboration and Teamwork: Group processes and team-related issues within the vendors’ organizations/stores.  General Attitude: How the interviewee thinks or feels about implementing WIC OS generally. |
| Context Characteristics | Leadership Support: Characteristics of leaders that either support or interfere with the implementation or sustainment of innovations.  Networks and Relationships: Formal or informal relationships that may be leveraged to support or that may hinder implementing WIC OS.  Structures and Systems: Formal or informal systems in which the organization is structured and managed and/or processes for accomplishing work.  Political Factors and Dynamic: Organizational politics that may positively or negatively affect decisions and activities related to implementation of WIC OS.  Organizational Priorities: Organizational policies, mandates, and/or priorities, whether or how these might support or hinder WIC OS implementation. |
| Facilitation Activities | Providing Education/Information - Educating Staff: Educating vendors’ staff on new skills related to WIC OS implementation.  Providing Education/Information - Marketing to Participants: Marketing materials for WIC OS to promote or publicize it.  Problem Identification and Resolution: Conducting or helping stakeholders identify implementation barriers and generate potential solutions to implementation barriers.  Providing Administrative/Technical Support: Conducting administrative tasks that support the implementation activities for WIC OS. |
